# *N*,*N*′-Bis(1-acetyl­cyclo­hexyl)-1,8:4,5-naphthalene­tetra­carboximide

**DOI:** 10.1107/S1600536809032206

**Published:** 2009-08-19

**Authors:** Chenaimwoyo A. Gondo, Daniel E. Lynch, Darren G. Hamilton

**Affiliations:** a Department of Chemistry, Mount Holyoke College, South Hadley, Masssachusetts 01075, USA; b Exilica Limited, The Technocentre, Puma Way, Coventry CV1 2TT, UK

## Abstract

The title compound, C_30_H_30_N_2_O_6_, has crystallographic inversion symmetry with the nitro­gen atom and the two oxygen atoms of the naphthalene diimide system deviating by −0.243 (2), 0.109 (3) and 0.247 (2) Å, respectively, from the plane defined by the carbon atoms.

## Related literature

For the structure of a related benzene diimide derivative with terminal acetyl­ene groups, see: Gondo *et al.* (2009 ▶). For preparative procedures for compounds of this type and for the title compound, see Hamilton *et al.* (1998 ▶, 1999 ▶); Raehm *et al.* (2002 ▶); Ahn *et al.* (1997 ▶).
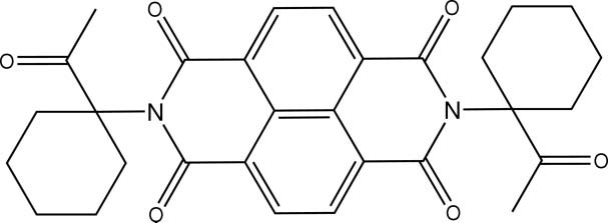


## Experimental

### 

#### Crystal data

C_30_H_30_N_2_O_6_
*M* *_r_* = 514.56
Monoclinic, 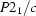

*a* = 5.8553 (2) Å
*b* = 13.6603 (6) Å
*c* = 15.2820 (6) Å
β = 94.001 (2)°
*V* = 1219.35 (8) Å^3^
*Z* = 2
Mo *K*α radiation
μ = 0.10 mm^−1^
*T* = 120 K
0.20 × 0.18 × 0.06 mm

#### Data collection

Bruker–Nonius 95 mm CCD camera on κ-goniostat diffractometer
Absorption correction: multi-scan (**SADABS**; Sheldrick, 2003 ▶) *T*_min_ = 0.981, *T*_max_ = 0.994
11932 measured reflections
2397 independent reflections
1949 reflections with *I* > 2σ(*I*)
*R*_int_ = 0.049

#### Refinement

*R*[*F* ^2^ > 2σ(*F* ^2^)] = 0.063
*wR*(*F* ^2^) = 0.150
*S* = 1.17
2397 reflections
174 parameters
H-atom parameters constrained
Δρ_max_ = 0.60 e Å^−3^
Δρ_min_ = −0.59 e Å^−3^

### 

Data collection: *COLLECT* (Hooft, 1998 ▶); cell refinement: *DENZO* (Otwinowski & Minor, 1997 ▶) and *COLLECT*; data reduction: *DENZO* and *COLLECT*; program(s) used to solve structure: *SHELXS97* (Sheldrick, 2008 ▶); program(s) used to refine structure: *SHELXL97* (Sheldrick, 2008 ▶); molecular graphics: *PLATON* (Spek, 2009 ▶); software used to prepare material for publication: *SHELXL97*.

## Supplementary Material

Crystal structure: contains datablocks I, global. DOI: 10.1107/S1600536809032206/zs2005sup1.cif

Structure factors: contains datablocks I. DOI: 10.1107/S1600536809032206/zs2005Isup2.hkl

Additional supplementary materials:  crystallographic information; 3D view; checkCIF report

## Figures and Tables

**Fig. 1.**
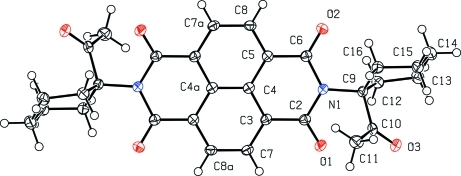
Molecular configuration and atom-numbering scheme for (I) which has inversion symetry (symmetry code: a -x + 1, -y + 1, -z + 1). Displacement ellipsoids are drawn at the 50% probability level.

## supplementary crystallographic information

### Comment

In a previous paper we presented the structure of a benzene diimide derivative having terminal acetylene groups and solubilizing cyclohexyl substituents (Gondo *et al.*, 2009). This material was prepared for use in oxidative coupling reactions, thereby forming macrocycles as either isolated entities (Hamilton *et al.*, 1999), or as components of molecularly interlocked systems (Hamilton *et al.*, 1998; Raehm *et al.*, 2002). As the corresponding naphthalene diimide analogues of benzene diimide derivatives are known to be generally more powerful electron acceptors, and have therefore been deployed in a variety of supramolecular and materials chemistry contexts, we attempted the preparation of the corresponding naphthalene diimide. However, under all of the standard conditions generally employed in the synthesis of benzene and naphthalene diimides we failed to obtain the desired compound. Only under rather forcing conditions was evidence of ring closure to the imide obtained, but under these conditions adventitious water was also found to have added to the acetylene groups (Ahn *et al.*, 1997). Thus, a low yield of the diketone was the only isolable material obtained from this process and the structure of this compound (I) is reported here.

The title compound has crystallographic inversion symmetry (Fig. 1), (symmetry code: a -*x* + 1, -*y* + 1, -*z* + 1). The nitrogen and the two oxygen atoms of the naphthalene diimide systems deviate by -0.243 (2), 0.109 (3) and 0.247 (2) Å respectively from the plane defined by the carbon atoms.

### Experimental

Under standard conditions for aromatic diimide formation (Hamilton *et al.*, 1998; Hamilton *et al.*, 1999) no evidence for the production of the desired acetylenic diimide could be found. Ring closure accompanied by unwanted addition of water across the acetylene bonds was observed using an alternative protocol (Ahn *et al.*, 1997), giving a very low yield (<5%) of diketone (I) after chromatographic isolation. Single crystals of suitable quality for structure determination were grown by vapor diffusion of water into a DMF solution of the title compound.

### Refinement

All H atoms were included in the refinement at calculated positions, in the riding-model approximation, with C–H distances of 0.95 (ArH), 0.98 (CH_3_) and 0.99Å (CH_2_). The isotropic displacement parameters for all H atoms were set equal to 1.25*U*_eq_ of the carrier atom. A large residual electron density (0.60 eÅ^-3^) is located 0.57Å from H4.

### Crystal data

**Table:**

C_30_H_30_N_2_O_6_	*F*(000) = 544
*M**_r_* = 514.56	*D*_x_ = 1.401 Mg m^−^^3^
Monoclinic, *P*2_1_/*c*	Mo *K*α radiation, λ = 0.71073 Å
Hall symbol: -P 2ybc	Cell parameters from 2881 reflections
*a* = 5.8553 (2) Å	θ = 2.9–27.5°
*b* = 13.6603 (6) Å	µ = 0.10 mm^−^^1^
*c* = 15.2820 (6) Å	*T* = 120 K
β = 94.001 (2)°	Plate, orange
*V* = 1219.35 (8) Å^3^	0.20 × 0.18 × 0.06 mm
*Z* = 2	

### Data collection

**Table:**

Bruker–Nonius 95 mm CCD camera on κ-goniostat diffractometer	2397 independent reflections
Radiation source: Bruker Nonius FR591 rotating anode	1949 reflections with *I* > 2σ(*I*)
10 cm confocal mirrors	*R*_int_ = 0.049
Detector resolution: 9.091 pixels mm^-1^	θ_max_ = 26.0°, θ_min_ = 3.1°
φ and ω scans	*h* = −7→7
Absorption correction: multi-scan (*SADABS*; Sheldrick, 2003)	*k* = −16→16
*T*_min_ = 0.981, *T*_max_ = 0.994	*l* = −18→16
11932 measured reflections	

### Refinement

**Table:**

Refinement on *F*^2^	Secondary atom site location: difference Fourier map
Least-squares matrix: full	Hydrogen site location: inferred from neighbouring sites
*R*[*F*^2^ > 2σ(*F*^2^)] = 0.063	H-atom parameters constrained
*wR*(*F*^2^) = 0.150	*w* = 1/[σ^2^(*F*_o_^2^) + (0.0786*P*)^2^ + 0.2672*P*] where *P* = (*F*_o_^2^ + 2*F*_c_^2^)/3
*S* = 1.17	(Δ/σ)_max_ < 0.001
2397 reflections	Δρ_max_ = 0.60 e Å^−^^3^
174 parameters	Δρ_min_ = −0.59 e Å^−^^3^
0 restraints	Extinction correction: *SHELXL97* (Sheldrick, 2008), Fc^*^=kFc[1+0.001xFc^2^λ^3^/sin(2θ)]^-1/4^
Primary atom site location: structure-invariant direct methods	Extinction coefficient: 0.094 (8)

### Special details

**Table:**

Experimental. The minimum and maximum absorption values stated above are those calculated in *SHELXL97* from the given crystal dimensions. The ratio of minimum to maximum apparent transmission was determined experimentally as 0.770335.
Geometry. Least-squares planes (*x*,*y*,*z* in crystal coordinates) and deviations from them (* indicates atom used to define plane) 3.5634 (0.0035) *x* - 0.8935 (0.0092) *y* + 11.4066 (0.0079) *z* = 7.0465 (0.0048) * -0.0167 (0.0010) C2 * 0.0088 (0.0016) C3 * -0.0098 (0.0013) C4 * -0.0071 (0.0012) C5 * 0.0161 (0.0011) C6 * 0.0087 (0.0011) C7 - 0.0027 (0.0028) C8 - 0.2429 (0.0022) N1 0.1089 (0.0026) O1 0.2471 (0.0023) O2 Rms deviation of fitted atoms = 0.0118

### Fractional atomic coordinates and isotropic or equivalent isotropic displacement parameters (Å^2^)

**Table:**

	*x*	*y*	*z*	*U*_iso_*/*U*_eq_	
O1	0.9857 (2)	0.37034 (9)	0.34838 (9)	0.0236 (4)	
O2	0.4959 (2)	0.19444 (9)	0.49973 (9)	0.0223 (4)	
O3	1.0165 (2)	0.19678 (10)	0.23338 (9)	0.0266 (4)	
N1	0.6940 (3)	0.28341 (11)	0.40186 (10)	0.0170 (4)	
C2	0.8167 (3)	0.37028 (13)	0.39014 (12)	0.0181 (4)	
C3	0.7265 (3)	0.46151 (13)	0.42772 (11)	0.0173 (4)	
C4	0.5438 (3)	0.45653 (13)	0.48277 (11)	0.0164 (4)	
C5	0.4497 (3)	0.36554 (13)	0.50527 (12)	0.0171 (4)	
C6	0.5449 (3)	0.27368 (13)	0.47037 (12)	0.0177 (4)	
C7	0.8165 (3)	0.55098 (13)	0.40659 (12)	0.0190 (4)	
H1	0.9401	0.5540	0.3695	0.024*	
C8	0.2735 (3)	0.36217 (13)	0.56043 (12)	0.0190 (4)	
H2	0.2125	0.3007	0.5763	0.024*	
C9	0.7659 (3)	0.19434 (13)	0.35194 (12)	0.0173 (4)	
C10	0.8453 (3)	0.22945 (13)	0.26254 (12)	0.0204 (5)	
C11	0.6832 (4)	0.29318 (15)	0.20654 (13)	0.0273 (5)	
H3	0.7698	0.3451	0.1795	0.034*	
H4	0.5707	0.3226	0.2432	0.034*	
H5	0.6039	0.2533	0.1606	0.034*	
C12	0.9518 (3)	0.13813 (13)	0.40723 (13)	0.0210 (5)	
H6	0.9015	0.1301	0.4673	0.026*	
H7	1.0936	0.1778	0.4115	0.026*	
C13	1.0070 (3)	0.03672 (14)	0.37068 (14)	0.0245 (5)	
H8	1.0946	0.0450	0.3180	0.031*	
H9	1.1052	0.0007	0.4151	0.031*	
C14	0.7927 (3)	−0.02394 (15)	0.34620 (15)	0.0278 (5)	
H10	0.8365	−0.0859	0.3182	0.035*	
H11	0.7153	−0.0404	0.3999	0.035*	
C15	0.6295 (3)	0.03341 (14)	0.28330 (13)	0.0234 (5)	
H12	0.4928	−0.0069	0.2668	0.029*	
H13	0.7062	0.0489	0.2292	0.029*	
C16	0.5565 (3)	0.12816 (13)	0.32632 (13)	0.0202 (5)	
H14	0.4763	0.1124	0.3795	0.025*	
H15	0.4483	0.1640	0.2852	0.025*	

### Atomic displacement parameters (Å^2^)

**Table:**

	*U*^11^	*U*^22^	*U*^33^	*U*^12^	*U*^13^	*U*^23^
O1	0.0219 (7)	0.0232 (7)	0.0269 (8)	−0.0025 (5)	0.0105 (6)	−0.0044 (6)
O2	0.0292 (8)	0.0171 (7)	0.0213 (7)	−0.0026 (6)	0.0057 (6)	0.0012 (5)
O3	0.0294 (8)	0.0235 (7)	0.0284 (8)	0.0017 (6)	0.0124 (6)	−0.0047 (6)
N1	0.0176 (8)	0.0171 (8)	0.0166 (8)	0.0003 (6)	0.0036 (6)	−0.0009 (6)
C2	0.0186 (9)	0.0210 (10)	0.0146 (9)	−0.0016 (7)	0.0007 (7)	−0.0005 (7)
C3	0.0182 (9)	0.0209 (10)	0.0125 (9)	−0.0015 (7)	−0.0003 (7)	−0.0020 (7)
C4	0.0149 (9)	0.0206 (10)	0.0132 (9)	−0.0001 (7)	−0.0016 (7)	−0.0001 (7)
C5	0.0184 (9)	0.0185 (10)	0.0140 (9)	−0.0003 (7)	−0.0013 (7)	−0.0001 (7)
C6	0.0178 (9)	0.0204 (10)	0.0147 (9)	−0.0015 (8)	−0.0003 (7)	−0.0010 (7)
C7	0.0197 (9)	0.0223 (10)	0.0153 (9)	−0.0015 (8)	0.0042 (7)	0.0004 (7)
C8	0.0218 (10)	0.0194 (9)	0.0160 (10)	−0.0039 (8)	0.0024 (7)	0.0016 (7)
C9	0.0163 (9)	0.0167 (9)	0.0192 (10)	0.0011 (7)	0.0032 (7)	−0.0016 (7)
C10	0.0240 (10)	0.0152 (9)	0.0223 (10)	−0.0024 (8)	0.0032 (8)	−0.0051 (7)
C11	0.0334 (12)	0.0279 (11)	0.0202 (10)	−0.0019 (9)	−0.0006 (9)	0.0019 (8)
C12	0.0189 (10)	0.0208 (10)	0.0232 (10)	0.0003 (8)	0.0007 (8)	0.0005 (8)
C13	0.0209 (10)	0.0213 (10)	0.0312 (11)	0.0041 (8)	0.0017 (8)	0.0028 (8)
C14	0.0257 (11)	0.0188 (10)	0.0397 (12)	0.0005 (8)	0.0074 (9)	−0.0013 (9)
C15	0.0212 (10)	0.0214 (10)	0.0280 (11)	−0.0043 (8)	0.0042 (8)	−0.0057 (8)
C16	0.0174 (9)	0.0210 (10)	0.0221 (10)	−0.0008 (8)	0.0013 (7)	−0.0020 (8)

### Geometric parameters (Å, °)

**Table:**

O1—C2	1.214 (2)	C9—C16	1.552 (3)
O2—C6	1.214 (2)	C10—C11	1.509 (3)
O3—C10	1.210 (2)	C11—H3	0.98
N1—C2	1.405 (2)	C11—H4	0.98
N1—C6	1.415 (2)	C11—H5	0.98
N1—C9	1.512 (2)	C12—C13	1.537 (3)
C2—C3	1.485 (3)	C12—H6	0.99
C3—C7	1.378 (3)	C12—H7	0.99
C3—C4	1.408 (3)	C13—C14	1.528 (3)
C4—C4^i^	1.410 (3)	C13—H8	0.99
C4—C5	1.412 (3)	C13—H9	0.99
C5—C8	1.378 (3)	C14—C15	1.524 (3)
C5—C6	1.487 (3)	C14—H10	0.99
C7—C8^i^	1.406 (3)	C14—H11	0.99
C7—H1	0.95	C15—C16	1.526 (3)
C8—C7^i^	1.406 (3)	C15—H12	0.99
C8—H2	0.95	C15—H13	0.99
C9—C12	1.536 (3)	C16—H14	0.99
C9—C10	1.550 (3)	C16—H15	0.99
			
C2—N1—C6	121.39 (15)	H3—C11—H4	109.5
C2—N1—C9	116.90 (14)	C10—C11—H5	109.5
C6—N1—C9	120.37 (14)	H3—C11—H5	109.5
O1—C2—N1	120.69 (16)	H4—C11—H5	109.5
O1—C2—C3	121.85 (16)	C9—C12—C13	114.19 (16)
N1—C2—C3	117.42 (16)	C9—C12—H6	108.7
C7—C3—C4	120.12 (16)	C13—C12—H6	108.7
C7—C3—C2	120.09 (16)	C9—C12—H7	108.7
C4—C3—C2	119.76 (16)	C13—C12—H7	108.7
C3—C4—C4^i^	119.6 (2)	H6—C12—H7	107.6
C3—C4—C5	120.91 (16)	C14—C13—C12	112.81 (16)
C4^i^—C4—C5	119.5 (2)	C14—C13—H8	109.0
C8—C5—C4	120.00 (16)	C12—C13—H8	109.0
C8—C5—C6	120.44 (16)	C14—C13—H9	109.0
C4—C5—C6	119.55 (16)	C12—C13—H9	109.0
O2—C6—N1	122.08 (16)	H8—C13—H9	107.8
O2—C6—C5	121.05 (16)	C15—C14—C13	110.10 (16)
N1—C6—C5	116.87 (15)	C15—C14—H10	109.6
C3—C7—C8^i^	120.33 (17)	C13—C14—H10	109.6
C3—C7—H1	119.8	C15—C14—H11	109.6
C8^i^—C7—H1	119.8	C13—C14—H11	109.6
C5—C8—C7^i^	120.45 (17)	H10—C14—H11	108.2
C5—C8—H2	119.8	C14—C15—C16	110.27 (16)
C7^i^—C8—H2	119.8	C14—C15—H12	109.6
N1—C9—C12	109.66 (14)	C16—C15—H12	109.6
N1—C9—C10	107.88 (14)	C14—C15—H13	109.6
C12—C9—C10	113.28 (15)	C16—C15—H13	109.6
N1—C9—C16	110.69 (14)	H12—C15—H13	108.1
C12—C9—C16	111.39 (15)	C15—C16—C9	111.25 (15)
C10—C9—C16	103.78 (15)	C15—C16—H14	109.4
O3—C10—C11	120.53 (17)	C9—C16—H14	109.4
O3—C10—C9	121.16 (17)	C15—C16—H15	109.4
C11—C10—C9	117.60 (16)	C9—C16—H15	109.4
C10—C11—H3	109.5	H14—C16—H15	108.0
C10—C11—H4	109.5		
			
C6—N1—C2—O1	160.09 (17)	C2—C3—C7—C8^i^	177.80 (17)
C9—N1—C2—O1	−6.8 (2)	C4—C5—C8—C7^i^	1.1 (3)
C6—N1—C2—C3	−22.2 (2)	C6—C5—C8—C7^i^	−179.98 (17)
C9—N1—C2—C3	170.95 (15)	C2—N1—C9—C12	89.38 (18)
O1—C2—C3—C7	8.2 (3)	C6—N1—C9—C12	−77.62 (19)
N1—C2—C3—C7	−169.49 (17)	C2—N1—C9—C10	−34.4 (2)
O1—C2—C3—C4	−173.83 (17)	C6—N1—C9—C10	158.59 (15)
N1—C2—C3—C4	8.5 (2)	C2—N1—C9—C16	−147.33 (16)
C7—C3—C4—C4^i^	−1.0 (3)	C6—N1—C9—C16	45.7 (2)
C2—C3—C4—C4^i^	−179.01 (19)	N1—C9—C10—O3	136.51 (17)
C7—C3—C4—C5	−179.86 (17)	C12—C9—C10—O3	14.9 (2)
C2—C3—C4—C5	2.2 (3)	C16—C9—C10—O3	−106.01 (19)
C3—C4—C5—C8	178.90 (16)	N1—C9—C10—C11	−53.1 (2)
C4^i^—C4—C5—C8	0.1 (3)	C12—C9—C10—C11	−174.65 (15)
C3—C4—C5—C6	0.0 (3)	C16—C9—C10—C11	64.39 (19)
C4^i^—C4—C5—C6	−178.82 (19)	N1—C9—C12—C13	169.46 (15)
C2—N1—C6—O2	−155.80 (17)	C10—C9—C12—C13	−70.0 (2)
C9—N1—C6—O2	10.6 (3)	C16—C9—C12—C13	46.6 (2)
C2—N1—C6—C5	24.2 (2)	C9—C12—C13—C14	−48.2 (2)
C9—N1—C6—C5	−169.37 (15)	C12—C13—C14—C15	54.2 (2)
C8—C5—C6—O2	−11.5 (3)	C13—C14—C15—C16	−60.5 (2)
C4—C5—C6—O2	167.34 (17)	C14—C15—C16—C9	60.2 (2)
C8—C5—C6—N1	168.43 (16)	N1—C9—C16—C15	−174.79 (15)
C4—C5—C6—N1	−12.7 (2)	C12—C9—C16—C15	−52.5 (2)
C4—C3—C7—C8^i^	−0.2 (3)	C10—C9—C16—C15	69.71 (18)

Symmetry codes: (i) −*x*+1, −*y*+1, −*z*+1.
